# 2-(Benzothia­zol-2-yl­sulfanyl)­acetic acid

**DOI:** 10.1107/S1600536810041796

**Published:** 2010-10-23

**Authors:** Zhi-li Fang, Jun Wang

**Affiliations:** a School of Basic Science, East China Jiaotong University, Nanchang 330013, People’s Republic of China; b Zhongshan Polytechnic, Zhongshan, Guangdong 528404, People’s Republic of China

## Abstract

In the title compound, C_9_H_7_NO_2_S_2_, the benzine ring is essentially co-planar with the thia­zole ring, making a dihedral angle of 0.36 (7)°. In the crystal structure, mol­ecules are linked by inter­molecular O—H⋯N hydrogen bonds between the carb­oxy group and the thia­zole N atom into chains along [10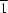]. The chains are assembled into a supermolecular layer structure by thia­zole ring S⋯S contacts [3.5679 (7) Å].

## Related literature

For the structure of tris­(2-hy­droxy­eth­yl)ammonium 3-benzo­thia­zole-2-thiol­ate, see: Zhu *et al.* (2009 ▶). For S⋯S contacts in similar compounds, see: Dai *et al.* (1997 ▶).
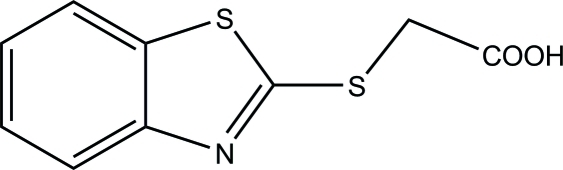


## Experimental

### 

#### Crystal data

C_9_H_7_NO_2_S_2_
*M* *_r_* = 225.28
Monoclinic, 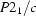

*a* = 6.0374 (5) Å
*b* = 19.2450 (17) Å
*c* = 8.1250 (7) Å
β = 90.419 (1)°
*V* = 944.02 (14) Å^3^
*Z* = 4
Mo *K*α radiation
μ = 0.53 mm^−1^
*T* = 296 K
0.23 × 0.21 × 0.17 mm

#### Data collection

Bruker APEXII CCD diffractometer
Absorption correction: multi-scan (*SADABS*; Sheldrick, 1996 ▶) *T*_min_ = 0.885, *T*_max_ = 0.914
4800 measured reflections
1695 independent reflections
1439 reflections with *I* > 2σ(*I*)
*R*_int_ = 0.021

#### Refinement

*R*[*F* ^2^ > 2σ(*F* ^2^)] = 0.030
*wR*(*F* ^2^) = 0.079
*S* = 1.04
1695 reflections
129 parameters
H-atom parameters constrained
Δρ_max_ = 0.25 e Å^−3^
Δρ_min_ = −0.16 e Å^−3^

### 

Data collection: *APEX2* (Bruker, 2004 ▶); cell refinement: *SAINT* (Bruker, 2004 ▶); data reduction: *SAINT*; program(s) used to solve structure: *SHELXS97* (Sheldrick, 2008 ▶); program(s) used to refine structure: *SHELXL97* (Sheldrick, 2008 ▶); molecular graphics: *SHELXTL* (Sheldrick, 2008 ▶); software used to prepare material for publication: *SHELXTL*.

## Supplementary Material

Crystal structure: contains datablocks I, global. DOI: 10.1107/S1600536810041796/ds2058sup1.cif

Structure factors: contains datablocks I. DOI: 10.1107/S1600536810041796/ds2058Isup2.hkl

Additional supplementary materials:  crystallographic information; 3D view; checkCIF report

## Figures and Tables

**Table 1:** Hydrogen-bond geometry (Å, °)

*D*—H⋯*A*	*D*—H	H⋯*A*	*D*⋯*A*	*D*—H⋯*A*
O1—H1⋯N1^i^	0.82	1.89	2.686 (2)	165

Symmetry code: (i) 
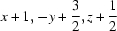
.

**Fig. 1.**
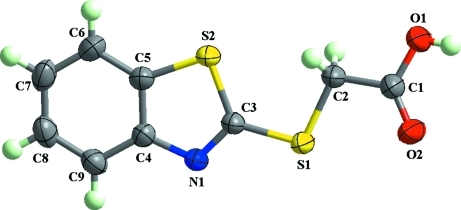
The structure of the title compound with 50% probability displacement ellipsoids.

**Fig. 2.**
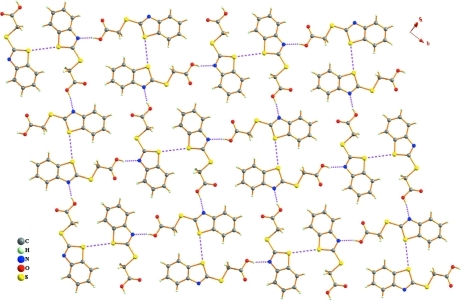
Two-dimensional supramolecular layer which is connected by O—H···N [O···N 2.686 (2) Å, H···N 1.89 Å, O—H···N 165.3°, symmetry code: x + 1, -y + 3/2,z + 1/2] hydrogen bonds and S···S [S···S 3.568 Å, symmetry code: 1-x, 1-y, 1-z] contacts.

## supplementary crystallographic information

### Experimental

A solution of benzothiazole-2-thiol (167.2 mg, 1.00 mmol) and K_2_CO_3 _(207.0 mg, 1.50 mmol) in CH_3_OH (15 ml) was slowly added to a solution of 2-chloroacetic acid (113.4 mg, 1.20 mmol) in CH_3_OH (10 ml). The resultant solution was stirred and refluxed for 20 h and then filtered. Colorless crystals suitable for X-ray diffraction were obtained in about a week by slow diffusion of diethyl ether into a dilute solution of the title compound in methanol. yield: *ca* 82.3% (based on benzothiazole-2-thiol).

### Refinement

The structure was solved using direct methods followed by Fourier synthesis. Non-H atoms were refined anisotropically. All of H atoms were placed in idealized positions (C—H = 0.93 or 0.97 Å, O—H = 0.82 Å), forced to ride on the atom to which they are bonded, and were included in the refinement in the riding-model approximation. *U*_iso_ values were set equal to 1.5*U*_eq_(parent atom) for carboxylic H atom and to 1.2*U*_eq_(parent atom)for all other H atoms.

### Crystal data

**Table:**

C_9_H_7_NO_2_S_2_	*F*(000) = 464
*M**_r_* = 225.28	*D*_x_ = 1.585 Mg m^−^^3^
Monoclinic, *P*2_1_/*c*	Mo *K*α radiation, λ = 0.71073 Å
Hall symbol: -P 2ybc	Cell parameters from 2101 reflections
*a* = 6.0374 (5) Å	θ = 2.7–27.5°
*b* = 19.2450 (17) Å	µ = 0.53 mm^−^^1^
*c* = 8.1250 (7) Å	*T* = 296 K
β = 90.419 (1)°	Block, pink
*V* = 944.02 (14) Å^3^	0.23 × 0.21 × 0.17 mm
*Z* = 4	

### Data collection

**Table:**

Bruker APEXII CCD diffractometer	1695 independent reflections
Radiation source: fine-focus sealed tube	1439 reflections with *I* > 2σ(*I*)
graphite	*R*_int_ = 0.021
φ and ω scans	θ_max_ = 25.2°, θ_min_ = 2.1°
Absorption correction: multi-scan (*SADABS*; Sheldrick, 1996)	*h* = −4→7
*T*_min_ = 0.885, *T*_max_ = 0.914	*k* = −22→23
4800 measured reflections	*l* = −9→9

### Refinement

**Table:**

Refinement on *F*^2^	Primary atom site location: structure-invariant direct methods
Least-squares matrix: full	Secondary atom site location: difference Fourier map
*R*[*F*^2^ > 2σ(*F*^2^)] = 0.030	Hydrogen site location: inferred from neighbouring sites
*wR*(*F*^2^) = 0.079	H-atom parameters constrained
*S* = 1.04	*w* = 1/[σ^2^(*F*_o_^2^) + (0.0386*P*)^2^ + 0.2852*P*] where *P* = (*F*_o_^2^ + 2*F*_c_^2^)/3
1695 reflections	(Δ/σ)_max_ = 0.001
129 parameters	Δρ_max_ = 0.25 e Å^−^^3^
0 restraints	Δρ_min_ = −0.16 e Å^−^^3^

### Special details

**Table:**

Geometry. All e.s.d.'s (except the e.s.d. in the dihedral angle between two l.s. planes) are estimated using the full covariance matrix. The cell e.s.d.'s are taken into account individually in the estimation of e.s.d.'s in distances, angles and torsion angles; correlations between e.s.d.'s in cell parameters are only used when they are defined by crystal symmetry. An approximate (isotropic) treatment of cell e.s.d.'s is used for estimating e.s.d.'s involving l.s. planes.
Refinement. Refinement of *F*^2^ against ALL reflections. The weighted *R*-factor *wR* and goodness of fit *S* are based on *F*^2^, conventional *R*-factors *R* are based on *F*, with *F* set to zero for negative *F*^2^. The threshold expression of *F*^2^ > σ(*F*^2^) is used only for calculating *R*-factors(gt) *etc*. and is not relevant to the choice of reflections for refinement. *R*-factors based on *F*^2^ are statistically about twice as large as those based on *F*, and *R*- factors based on ALL data will be even larger.

### Fractional atomic coordinates and isotropic or equivalent isotropic displacement parameters (Å^2^)

**Table:**

	*x*	*y*	*z*	*U*_iso_*/*U*_eq_	
C1	0.5666 (3)	0.77360 (10)	0.5938 (3)	0.0446 (5)	
C2	0.4631 (3)	0.70448 (10)	0.5538 (3)	0.0424 (5)	
H2A	0.4289	0.6798	0.6546	0.051*	
H2B	0.5654	0.6764	0.4906	0.051*	
C3	0.1497 (3)	0.63490 (9)	0.3747 (2)	0.0367 (4)	
C4	−0.0451 (3)	0.55193 (10)	0.2515 (2)	0.0371 (4)	
C5	0.1331 (3)	0.51163 (10)	0.3081 (2)	0.0391 (4)	
C6	0.1436 (4)	0.44073 (10)	0.2773 (3)	0.0509 (5)	
H6	0.2624	0.4143	0.3154	0.061*	
C7	−0.0259 (4)	0.41063 (11)	0.1894 (3)	0.0556 (6)	
H7	−0.0223	0.3632	0.1677	0.067*	
C8	−0.2024 (4)	0.45021 (12)	0.1327 (3)	0.0531 (6)	
H8	−0.3154	0.4287	0.0732	0.064*	
C9	−0.2144 (3)	0.52022 (11)	0.1620 (2)	0.0458 (5)	
H9	−0.3337	0.5461	0.1228	0.055*	
N1	−0.0309 (3)	0.62213 (8)	0.2909 (2)	0.0396 (4)	
O1	0.7348 (3)	0.76603 (7)	0.6956 (2)	0.0557 (4)	
H1	0.7911	0.8041	0.7140	0.084*	
O2	0.5039 (3)	0.82751 (8)	0.5396 (2)	0.0736 (5)	
S1	0.21328 (9)	0.71923 (3)	0.43642 (7)	0.04900 (19)	
S2	0.31920 (8)	0.56396 (2)	0.41411 (7)	0.04382 (18)	

### Atomic displacement parameters (Å^2^)

**Table:**

	*U*^11^	*U*^22^	*U*^33^	*U*^12^	*U*^13^	*U*^23^
C1	0.0430 (11)	0.0378 (11)	0.0528 (12)	0.0004 (9)	−0.0105 (10)	−0.0021 (9)
C2	0.0413 (11)	0.0343 (10)	0.0515 (12)	0.0031 (8)	−0.0147 (9)	0.0004 (8)
C3	0.0340 (10)	0.0336 (10)	0.0422 (11)	0.0021 (8)	−0.0077 (8)	0.0014 (8)
C4	0.0402 (10)	0.0332 (10)	0.0378 (10)	−0.0017 (8)	−0.0033 (8)	0.0011 (8)
C5	0.0420 (11)	0.0354 (10)	0.0398 (10)	0.0014 (8)	−0.0068 (9)	0.0023 (8)
C6	0.0621 (14)	0.0344 (11)	0.0560 (13)	0.0066 (9)	−0.0087 (11)	0.0005 (9)
C7	0.0772 (16)	0.0335 (11)	0.0562 (13)	−0.0083 (11)	−0.0032 (12)	−0.0042 (10)
C8	0.0589 (14)	0.0514 (13)	0.0489 (12)	−0.0166 (11)	−0.0080 (10)	−0.0046 (10)
C9	0.0410 (11)	0.0495 (12)	0.0468 (12)	−0.0027 (9)	−0.0099 (9)	0.0009 (9)
N1	0.0387 (9)	0.0344 (8)	0.0455 (9)	0.0022 (7)	−0.0121 (7)	0.0010 (7)
O1	0.0507 (9)	0.0409 (8)	0.0751 (11)	−0.0069 (7)	−0.0270 (8)	0.0023 (7)
O2	0.0777 (12)	0.0336 (9)	0.1090 (14)	−0.0015 (8)	−0.0464 (10)	0.0066 (8)
S1	0.0454 (3)	0.0309 (3)	0.0703 (4)	0.0053 (2)	−0.0231 (3)	−0.0041 (2)
S2	0.0415 (3)	0.0336 (3)	0.0560 (3)	0.00667 (19)	−0.0177 (2)	−0.0010 (2)

### Geometric parameters (Å, °)

**Table:**

C1—O2	1.188 (2)	C4—C5	1.401 (3)
C1—O1	1.313 (2)	C5—C6	1.389 (3)
C1—C2	1.504 (3)	C5—S2	1.7332 (19)
C2—S1	1.8012 (19)	C6—C7	1.372 (3)
C2—H2A	0.9700	C6—H6	0.9300
C2—H2B	0.9700	C7—C8	1.386 (3)
C3—N1	1.304 (2)	C7—H7	0.9300
C3—S2	1.7345 (18)	C8—C9	1.370 (3)
C3—S1	1.7407 (18)	C8—H8	0.9300
C4—N1	1.391 (2)	C9—H9	0.9300
C4—C9	1.391 (3)	O1—H1	0.8200
			
O2—C1—O1	124.99 (19)	C4—C5—S2	109.55 (14)
O2—C1—C2	124.15 (19)	C7—C6—C5	118.3 (2)
O1—C1—C2	110.86 (17)	C7—C6—H6	120.9
C1—C2—S1	108.66 (14)	C5—C6—H6	120.9
C1—C2—H2A	110.0	C6—C7—C8	120.7 (2)
S1—C2—H2A	110.0	C6—C7—H7	119.7
C1—C2—H2B	110.0	C8—C7—H7	119.7
S1—C2—H2B	110.0	C9—C8—C7	121.6 (2)
H2A—C2—H2B	108.3	C9—C8—H8	119.2
N1—C3—S2	115.97 (14)	C7—C8—H8	119.2
N1—C3—S1	120.51 (14)	C8—C9—C4	118.9 (2)
S2—C3—S1	123.51 (11)	C8—C9—H9	120.6
N1—C4—C9	126.16 (18)	C4—C9—H9	120.6
N1—C4—C5	114.61 (16)	C3—N1—C4	110.65 (15)
C9—C4—C5	119.22 (18)	C1—O1—H1	109.5
C6—C5—C4	121.37 (18)	C3—S1—C2	100.82 (9)
C6—C5—S2	129.08 (16)	C5—S2—C3	89.21 (9)
			
O2—C1—C2—S1	−7.7 (3)	C5—C4—C9—C8	0.4 (3)
O1—C1—C2—S1	172.54 (15)	S2—C3—N1—C4	0.0 (2)
N1—C4—C5—C6	−179.50 (19)	S1—C3—N1—C4	178.49 (14)
C9—C4—C5—C6	−0.3 (3)	C9—C4—N1—C3	−179.45 (19)
N1—C4—C5—S2	0.5 (2)	C5—C4—N1—C3	−0.3 (2)
C9—C4—C5—S2	179.69 (15)	N1—C3—S1—C2	176.73 (16)
C4—C5—C6—C7	0.0 (3)	S2—C3—S1—C2	−4.87 (16)
S2—C5—C6—C7	−179.96 (17)	C1—C2—S1—C3	170.23 (15)
C5—C6—C7—C8	0.2 (3)	C6—C5—S2—C3	179.6 (2)
C6—C7—C8—C9	−0.1 (4)	C4—C5—S2—C3	−0.40 (15)
C7—C8—C9—C4	−0.2 (3)	N1—C3—S2—C5	0.25 (16)
N1—C4—C9—C8	179.49 (19)	S1—C3—S2—C5	−178.21 (14)

### Hydrogen-bond geometry (Å, °)

**Table:**

*D*—H···*A*	*D*—H	H···*A*	*D*···*A*	*D*—H···*A*
O1—H1···N1^i^	0.82	1.89	2.686 (2)	165

Symmetry codes: (i) *x*+1, −*y*+3/2, *z*+1/2.
